# Genetic and Clinical Characteristics of Thalassemia Trait and Construction of LASSO‐Based Model for Its Differentiating From Iron Deficiency Anemia in Northern China

**DOI:** 10.1002/jcla.70386

**Published:** 2026-10-26

**Authors:** Wen‐jie Yu, Feng‐qi Zhang, Jia‐feng Niu, Rui Ma, Yin‐ting Ma, Guang‐ping Li, Yan Long

**Affiliations:** ^1^ Department of Clinical Laboratory Peking University People's Hospital Beijing China; ^2^ Department of Clinical Laboratory Tangshan Workers' Hospital Tangshan Hebei China; ^3^ Department of Clinical Laboratory Sanmenxia Central Hospital Sanmenxia Henan China; ^4^ Department of Clinical Laboratory Qingdao Chengyang District People's Hospital Qingdao Shandong China

**Keywords:** genotype distribution, iron deficiency anemia, nomogram, thalassemia

## Abstract

**Background:**

Thalassemia trait (TT) and iron deficiency anemia (IDA) represent the most common causes of microcytic hypochromic anemia (MHA) and are challenging to differentiate in clinical settings. Data regarding the genetic and clinical characteristics of TT in Northern China remain insufficient, highlighting an urgent need for rapid and efficient diagnostic strategies for the regional population.

**Methods:**

This retrospective study enrolled 482 TT and 640 IDA patients from Northern China. Twenty‐seven α‐ and β‐TT mutations were detected by multicolor probe melting curve analysis (MMCA) and further confirmed by Sanger sequencing. A LASSO regression model using routine hematological indices was constructed and further validated. The diagnostic efficacy was compared with other indices (Mentzer, Shine and Lal, and Green and King indices).

**Results:**

A total of 482 cases with TT mutations were identified. β‐TT mutations were the most prevalent (*n* = 261, 54.15%), followed by α‐TT mutations (*n* = 209, 43.36%) and α+β complex TT mutations (*n* = 12, 2.49%). Among which, eight α‐TT genotypes, eleven β‐TT genotypes, and eight α+β compound genotypes mutations were detected. For α‐TT, the predominant genotype was *_α* ^*SEA*^ */αα* (77.0%), followed by *_α* ^*3.7*^ */αα* (14.3%), *α* ^*SEA*^ */_α* ^*3.7*^ (3.3%) and the rare *HKαα* (1.0%). The three most prevalent genotypes among β‐TT mutations were *HBB:c.316_197C>T heterozygous* (*het*) (35.2%), *HBB:c.124_127delTTCT het* (29.5%), and *HBB:c.52A>T het* (23.4%). α‐TT minor (33.82%) and β‐TT minor (53.94%) contributed the predominant phenotypes. The LASSO‐nomogram based on RBC, MCV, MCHC, and RDW‐SD achieved favorable discriminative performance (AUC = 0.94, sensitivity = 90.0%, specificity = 91.8%), showing superior diagnostic efficacy than other indices.

**Conclusion:**

Our study revealed a distinct β‐TT‐dominant genotypic profile in Northern China and provided a simple, region‐adapted model as a potential tool for differentiating TT from IDA in clinical practice.

## Graphical abstract

This study revealed a β‐thalassemia‐trait‐dominated genetic spectrum in Northern‐Chinese patients with microcytic hypochromic anemia, with *_α* ^*SEA*^ */αα*, *HBB*:c.316_197C>T and *HBB*:c.124_127delTTCT being the most prevalent α‐ and β‐globin genotypes. Using LASSO regression, we constructed a nomogram based on four routine complete‐blood‐count parameters (RBC, MCV, MCHC, RDW‐SD) to differentiate thalassemia trait from iron deficiency anemia. The nomogram achieved robust internal‐validation performance (test‐set AUC = 0.941) and outperformed traditional hematological indices, offering a convenient region‐adapted screening tool for clinical practice.

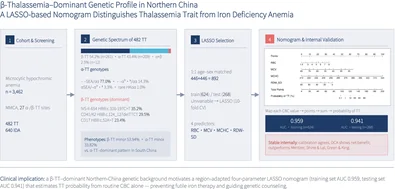

## 1 Introduction

Thalassemia trait (TT) and iron deficiency anemia (IDA) are two common types of microcytic hypochromic anemia (MHA) presented in clinical practice [1, 2]. TT is a hereditary hemolytic anemia resulting from reduced or absent synthesis of globin chains caused by mutations in globin genes [2, 3, 4, 5, 6, 7], among which α‐thalassemia (α‐TT) and β‐thalassemia (β‐TT) are the most common types in clinical practice [8]. IDA, in contrast, is caused by inadequate iron availability for hemoglobin synthesis, typically resulting from insufficient dietary intake, chronic blood loss, or impaired iron absorption [9]. TT was previously reported to occur mainly in Southern China but was rarely found in Northern China [5, 7, 10], data on the genotype distribution of TT in Northern China populations remain limited.

In clinical practice, it is frequently difficult to distinguish IDA from TT due to their similar clinical manifestations. However, the treatment strategies for TT or IDA differ fundamentally, and inappropriate treatment may lead to iron overload in patients with TT or delayed iron supplementation in those with IDA [3]. Therefore, accurate and timely discrimination between TT and IDA is essential for appropriate clinical treatment and patient outcomes. The conventional discrimination methods include complete blood counts (CBCs) test, hemoglobin electrophoresis, iron metabolism indices, and genetic detection [11, 12]. However, these methods require frequent blood sampling, increased testing costs, long turnaround times, and demand specialized expertise from clinicians and laboratory staff [13]. Meanwhile, some techniques, such as genetic testing, are equipment‐intensive and unsuitable for rapid diagnosis, limiting their application in resource‐limited or community‐based settings [2]. Therefore, a rapid, accurate, and clinically practical method is urgently needed to effectively discriminate TT from IDA in routine clinical settings.

In our current study, we aimed to systematically investigate the genetic and clinical distribution characteristics of TT in Northern China. We further sought to establish a prediction model based on erythrocyte parameters for accurate differentiation of TT from IDA, which may reduce the waiting times and financial burdens and facilitate timely treatment.

## 2 Methods and Materials

### 2.1 Study Subjects

We conducted a retrospective observational study that consecutively enrolled 3462 patients diagnosed with microcytic hypochromic anemia (MHA) at Peking University People's Hospital from January 2023 to December 2025. All participants underwent serum ferritin detection and full α/β‐globin gene mutation screening. The primary cohort included 482 TT carriers and 640 cases of IDA patients. To attenuate confounding factors, IDA patients were randomly sampled from all eligible IDA subjects and matched with TT by age and sex by a ratio of 1:1, thus a total of 446 matched TT‐IDA pairs was used for subsequent modeling. All matched subjects were randomly split into a training cohort (70%, 312 pairs) and an internal testing cohort (30%, 134 pairs), as illustrated in Figure 1. The enrollment criteria for MHA patients were as follows: (1) age ≥ 18 years. (2) Hb ≤ 110 g/L, MCV < 82 fL and MCH < 27 pg [14]. (3) Patients with complete clinical data. Exclusion criteria included: (1) Concurrent acute or chronic inflammatory disease, malignant diseases, other hematological diseases or tumors (leukemia, megaloblastic anemia and autoimmune hemolytic anemia etc.). (2) History of blood transfusion within the past 3 months. (3) Oral or intravenous iron supplementation within last 3 months. (4) Patients with concurrent TT and IDA. IDA was diagnosed according to the criteria as follows: (1) CBCs presented as the characteristics of MHA changes. (2) Serum ferritin < 30 μg/L [15]. This study was approved by the Medical Ethics Committee of the Peking University People's Hospital, and written informed consent was obtained from all participants.

**FIGURE 1:**
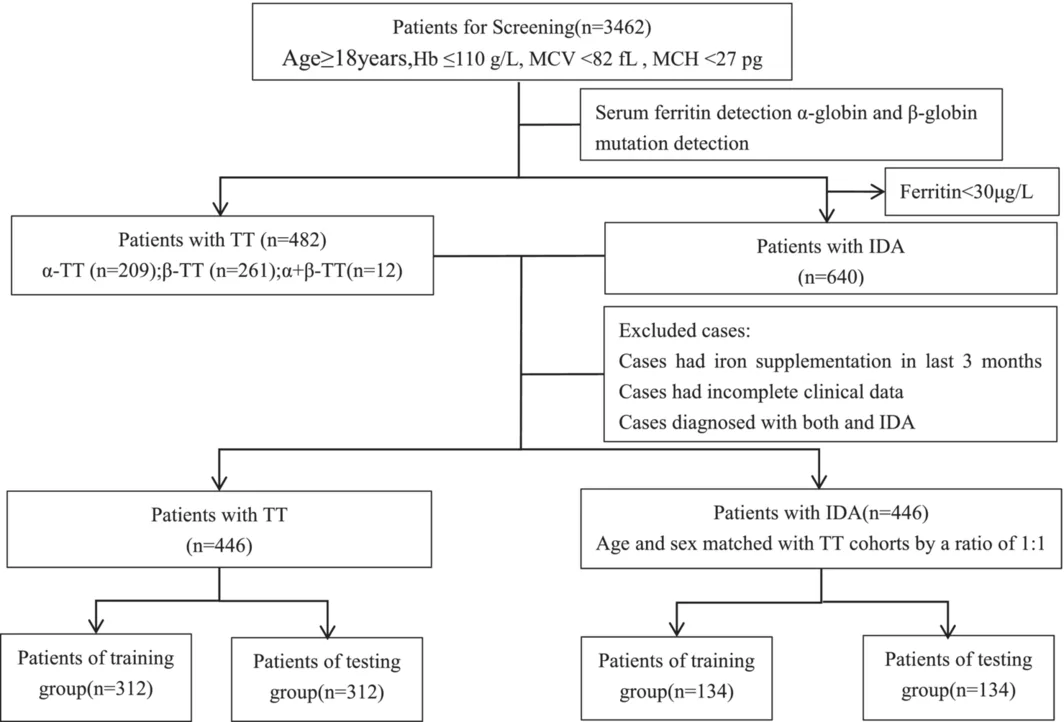
Flowchart of patient selection and cohort assignment. A total of 3462 patients diagnosed with MHA were initially screened. All patients underwent serum ferritin detection and α/β‐globin gene mutation analysis. Individuals were excluded if they had received iron supplementation within the previous 3 months, had incomplete clinical data, or presented with concurrent TT and IDA. After screening, 482 TT carriers and 640 IDA patients were retained, and subsequently 446 TT and 446 IDA patients were included for final model construction and validation by 1:1 age‐ and sex‐matching. The enrolled subjects were further randomly divided into a training cohort (*n* = 624, 312 TT and 312 IDA) and a testing cohort (*n* = 268, 134 TT and 134 IDA) at a ratio of 7:3. IDA, iron deficiency anemia; MHA, microcytic hypochromic anemia; TT, thalassemia trait.

### 2.2 Clinical Data Measurements and Collections

Routine CBC were detected using the Sysmex XE‐2100 analyzer (TOA Medical Electronics, Kobe, Japan). Serum ferritin concentrations were measured by Roche electrochemiluminescence immunoassay (ECLIA) analyzer (Roche Pharma. Switzerland). Clinical laboratory indicators were collected, including red blood cell count (RBC), hemoglobin (Hb), hematocrit (HCT), mean corpuscular hemoglobin (MCH), mean corpuscular hemoglobin concentration (MCHC), mean corpuscular volume (MCV), red blood cell distribution width coefficient of variation (RDW‐CV), red blood cell distribution width standard deviation (RDW‐SD), and serum ferritin.

### 2.3 DNA Extraction and Genetic Mutation Analysis

EDTA‐K_2_ anticoagulated peripheral blood samples were collected from all participants for genomic deoxyribonucleic acid (DNA) extractions. DNA was extracted from peripheral blood mononuclear cells (PBMCs) using a commercial DNA extraction kit (Tian‐Long Biotechnology Co. Ltd.) according to the manufacturer's instructions, and the purity and concentration were assessed using a NanoDrop spectrophotometer (Thermo Fisher Scientific), and extracted DNA was stored at −80°C until further detections.

A multicolor probe melting‐curve analysis (MMCA) panel was applied to detect the most prevalent α‐ and β‐TT mutations reported in the Chinese population. This panel covered three common α‐TT deletions (_*SEA*, _*α* ^*3.7*^ and _*α* ^*4.2*^), three α‐TT single nucleotide variants (*369C>G*, *427T>C* and *377T>C*), and 21 β‐TT gene mutations (*c.‐78A>G, c.79A>G, c.‐80T>C, c.‐81A>C*, *c.‐82C>A*, *c.‐123A>T*, *c.140C>T*, *c.79G>A*, *c.92+5G>C*, *c.315+5G>C*, *c.316_197C>T, c.91A>G*, *c.92+1G>T*, *c.45_46insG*, *c.48_49insG*, *c.52A>T*, *c.84_85insC*, *c.124_127delTTC*, *c.113G>A*, *c.130G>T, c.216_217insA*).

PCR amplification was carried out on a SLAN‐96S real‐time PCR system (Hong‐Shi Medical Technology Co. Ltd., Shanghai, China). Melting‐curve fluorescence signals were continuously acquired at 0.4°C intervals over a temperature range of 40°C–85°C. All mutation signals identified by MMCA were further validated by Sanger sequencing to guarantee detection accuracy. All detected genetic variants were annotated according to the standard HGVS nomenclature.

### 2.4 Statistical Analysis

All analyses were performed using R software (version 4.5.2). In the training set, univariable logistic regression was first applied to screen hematological parameters associated with TT, then statistically significant parameters were subsequently entered into the LASSO regression to mitigate collinearity and select the most informative predictors. The 1‐SE rule (*λ*.1se) was used to select the penalty parameter, yielding a parsimonious set of predictor variables. A multivariable logistic regression model was subsequently fitted, and a nomogram was constructed to facilitate intuitive individual‐level risk prediction.

An independent testing set was employed for internal validation using calibration curves. The AUC, sensitivity, specificity, PPV, and NPV at the optimal cutoff derived from the Youden index were calculated to evaluate the discriminative efficiency of the model. Decision‐curve analysis (DCA) was performed to assess whether the nomogram provided net clinical benefit across a range of threshold probabilities.

To evaluate the performance of our predictive model, its AUC was compared with those of three classic indices (Mentzer, Shine and Lal, and Green and King) using the same testing dataset. All statistical analyses were implemented in R with core packages, including readxl, dplyr, tidyr, glmnet, logistf, ggplot2, broom, scales, gridExtra, grid, pROC, patchwork, and rms.

## 3 Results

### 3.1 Genetic and Clinical Characteristics of TT Patients

The MMCA assay was used to screen 27 TT mutation sites covering the most common α‐TT and β‐TT mutations reported in the Chinese population [16]. Among which, 209 (43.36%) cases with α‐TT mutations, 261 (54.15%) cases with β‐TT mutations, and 12 (2.49%) patients with compound α+β‐TT mutations were identified, see in Table 1.

**TABLE 1:** Genotypes distributions of TT patients in Northern China.

Categories	Genotypes	Cases, *n* (%)
α‐TT	*_α* ^*SEA*^ */αα*	161 (77.0)
	*_α* ^*3.7*^ */αα*	30 (14.3)
	*_α* ^*SEA*^ */_α* ^*3.7*^	7 (3.3)
	*_α* ^*4.2*^ */αα*	4 (1.9)
	*_α* ^*SEA*^ */_α* ^*4.2*^	2 (1.0)
	*_α* ^*3.7*^ */_α* ^*3.7*^	2 (1.0)
	*HBA:c.427T>C het*	1 (0.5)
	*HKαα*	2 (1.0)
Total (α‐TT)		209
β‐TT	*HBB:c.316_197C>T het*	92 (35.2)
	*HBB:c.124_127delTTCT het*	77 (29.5)
	*HBB:c.52A>T het*	61 (23.4)
	*HBB:c.‐78A>G het*	8 (3.1)
	*HBB:c.84_85insC het*	6 (2.3)
	*HBB:c.216_217insA het*	6 (2.3)
	*HBB:c.130G>T het*	5 (1.9)
	*HBB:c.113G>A het*	3 (1.1)
	*HBB:c.‐80C>G het*	1 (0.4)
	*HBB:c.92+1G>T het*	1 (0.4)
	*HBB:c.42_46insG het*	1 (0.4)
Total (β‐TT)		261
α+β‐TT compound	*_α* ^*3.7*^ */αα, HBB:c.316_197C>T het*	3 (25.0)
	*_α* ^*4.2*^ */αα, HBB:c.124_127delTTCT het*	2 (16.7)
	*_α* ^*SEA*^ */αα, HBB:c.52A>T het*	2 (16.7)
	*_α* ^*3.7*^ */αα, HBB:c.79G>A het*	1 (8.3)
	*_α* ^*3.7*^ */αα, HBB:c.130G>T het*	1 (8.3)
	*_α* ^*SEA*^ */αα, HBB:c.‐78A>G het*	1 (8.3)
	*_α* ^*3.7*^ */αα, HBB:c.52A>T het*	1 (8.3)
	*HBB*: *c.369C>G het, HBB: c.123_126delTTCT het*	1 (8.3)
Total (α+β‐TT *het* compound)		12

*Note:* α‐globin gene deletions were presented as conventional nomenclature, β‐TT genetic variants detected were presented according to the standard HGVS nomenclature.

Abbreviations: Hb H disease, hemoglobin H disease; het, heterozygous; α‐TT, α‐thalassemia trait; β‐TT, β‐thalassemia trait.

Eight distinct genotypes were detected within α‐TT subgroup. Among which, the most prevalent genotype detected was *_α* ^*SEA*^ */αα* (77.0%), followed by *_α* ^*3.7*^ */αα* (14.3%) and *_α* ^*SEA*^ */_α* ^*3.7*^ (3.3%). Two cases carrying *HKαα* were also identified. Among patients with β‐TT, 11 genotypes were observed. *HBB:c.316‐197C>T heterozygous*(*het*) (35.2%) was the most prevalent genetype, followed by *HBB:c.124_127delTTCT het* (29.5%) and *HBB:c.52A>T het* (23.4%). Additionally, eight genotypes within 12 patients with compound α+β‐TT mutations were identified, among whom the most common genotype was *_α* ^*3.7*^ */αα* combined with *HBB:c.316‐197C>T het* (25.0%), Table 1. All identified TT genotype mutations in our enrolled subjects were heterozygous, with no homozygous variants were detected in this study cohort.

Regarding the clinical phenotypes of patients with α‐TT, α‐TT minor was the most common clinical phenotype, accounting for 163 cases (33.82%), followed by α‐TT silent (37 cases, 7.68%). Notably, nine cases (1.87%) of Hb H disease were identified among patients with α‐TT. For patients with β‐TT, β‐TT minor was the overwhelmingly predominant phenotype, presenting in 260 patients (53.94%), whereas β‐TT major was rare with only one case (0.21%) detected. Twelve cases with concurrent α‐TT and β‐TT were also observed. Among which, the combination of α‐TT silent with β‐TT minor was more prevalent (8 cases, 1.66%) than the combination of α‐TT minor with β‐TT minor (4 cases, 0.83%), Table 2.

**TABLE 2:** Phenotypic distributions of TT patients.

Phenotype	Cases, *n* (%)
α‐TT	
α‐TT minor	163 (33.82)
α‐TT silent	37 (7.68)
Hb H disease	9 (1.87)
Subtotal	209 (43.36)
β‐TT	
β‐TT minor	260 (53.94)
β‐TT major	1 (0.21)
Subtotal	261 (54.15)
α+β compound TT	
α‐TT silent with β‐TT minor	8 (1.66)
α‐TT minor with β‐TT minor	4 (0.83)
Subtotal	12 (2.49)
Total	482 (100)

Abbreviations: Hb H disease, hemoglobin H disease; α‐TT, α‐thalassemia trait; β‐TT, β‐thalassemia trait.

### 3.2 Baseline Characteristics of Patients for Model Construction

In our study, we performed 1:1 age‐sex matching to finally include 446 patients with TT and 446 patients with IDA from the initially recruited subjects. The matched participants were subsequently randomly divided into training and testing sets at a ratio of 7:3, yielding 312 pairs (312 TT and 312 IDA) for the training set and 134 pairs (134 TT and 134 IDA) for the testing set, Figure 1.

Baseline characteristics of the 892 enrolled patients were summarized in Table 3. Compared with IDA patients, patients with TT exhibited significantly higher RBC count, Hb, HCT, and MCHC, whereas MCV, RDW‐CV, and RDW‐SD were markedly lower Tables S1 and S2.

**TABLE 3:** Baseline characteristics in training and validation cohorts.

Variable	Total (*n* = 892)	TT (*n* = 446)	IDA (*n* = 446)	*p*
Age, median (Q1, Q3)	33.0 (28.0, 41.0)	33.0 (28.0, 41.3)	32.5 (28.0, 41.3)	0.069
RBC (10^12^/L), median (Q1, Q3)	4.8 (4.3, 5.6)	5.50 (4.9, 6.1)	4.4 (4.0, 4.8)	< 0.001
Hb (g/L), median (Q1, Q3)	103.0 (87.0, 116.0)	113.0 (102.0,126.0)	92.0 (75.0,103.0)	< 0.001
HCT (%), median (Q1, Q3)	34.1 (30.0, 37.9)	36.7 (32.8, 40.5)	31.7 (27.7, 35.0)	< 0.001
MCV (fl), median (Q1, Q3)	68.9 (64.9, 73.9)	66.9 (63.8, 70.0)	71.8 (67.2, 76.0)	< 0.001
MCH (pg), median (Q1, Q3)	20.6 (19.3, 22.0)	20.5 (19.6, 21.5)	20.8 (18.4, 22.8)	0.286
MCHC (g/L), median (Q1, Q3)	301.0 (288.0, 310.0)	308.0 (301.8, 312.0)	289.0 (273.0, 299.0)	< 0.001
RDW‐CV (%), median (Q1, Q3)	16.9 (15.5, 18.6)	16.1 (15.1, 17.4)	17.7 (16.2, 19.9)	< 0.001
RDW‐SD (fL), median (Q1, Q3)	40.8 (36.0, 45.2)	36.1 (34.5, 38.4)	44.7 (42.2, 47.8)	< 0.001

*Note:* Continuous variables were presented as median (Q1, Q3) and compared between TT and IDA groups using the Mann–Whitney *U* test. A two‐tailed *p* < 0.05 was considered statistically significant.

Abbreviations: HB, hemoglobin; HCT, hematocrit; IDA, iron deficient anemia; MCH, mean corpuscular hemoglobin; MCHC, mean corpuscular hemoglobin concentration; MCV, mean corpuscular volume; RBC, red blood cell; RDW‐CV, red blood cell distribution width coefficient of variation; RDW‐SD, red blood cell distribution width standard deviation; TT, thalassemia trait.

### 3.3 Variables Selection, Model Construction and Internal Validation

In the univariable logistic regression analysis, seven hematological parameters were significantly associated with TT in the training cohort (*p* < 0.001). RBC count (OR = 5.846, 95% CI: 4.393–7.977), HCT (OR = 1.190, 95% CI: 1.150–1.234), MCHC (OR = 1.150, 95% CI: 1.124–1.179) and Hb (OR = 1.071, 95% CI: 1.059–1.085) exhibited significant positive associations with TT, whereas RDW‐SD (OR = 0.715, 95% CI: 0.678–0.751), RDW‐CV (OR = 0.719, 95% CI: 0.662–0.779), and MCV (OR = 0.902, 95% CI: 0.875–0.928) were inversely associated with TT, Figure 2. These findings indicated that erythrocyte‐related parameters presented distinct distribution patterns between TT and IDA, supporting their potential utility for the differential diagnosis of TT and IDA.

**FIGURE 2:**
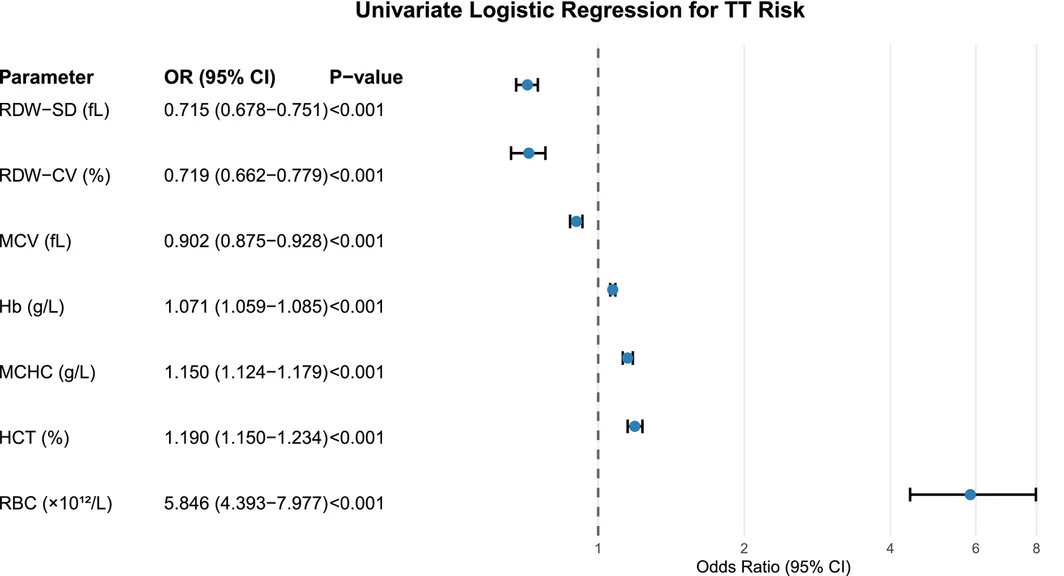
Univariate logistic regression analysis of hematological parameters associated with TT. Forest plot displaying ORs with 95% CIs for each parameter in the training cohort. The *x*‐axis was presented on a logarithmic scale, and the dashed vertical line marked OR = 1 (no effect). Parameters with OR > 1 (RBC, HCT, MCHC and Hb) indicate positive associations with TT, whereas those with OR < 1 (MCV, RDW‐CV, and RDW‐SD) indicated inverse associations. All displayed parameters achieved *p* < 0.001. CIs, confidence intervals; Hb, hemoglobin; HCT, hematocrit; MCHC, mean corpuscular hemoglobin concentration; MCV, mean corpuscular volume; OR, odds ratio; RBC, red blood cell count; RDW‐CV, red blood cell distribution width‐coefficient of variation; RDW‐SD, red blood cell distribution width‐standard deviation; TT, thalassemia trait.

To further optimize variable selection and alleviate overfitting, LASSO regression with 10‐fold cross‐validation was adopted for predictor screening. This approach could effectively balance the inherent bias‐variance trade‐off in model construction and has been proven to yield stable and robust performance across various predictive modeling scenarios [17, 18]. As depicted in Figure 3B, model performance varied with the penalty parameter *λ*. Specifically, larger *λ* values imposed stronger regularization, which progressively shrinks regression coefficients toward zero. The vertical dashed lines indicated *λ*.min = 0.00452 (minimum deviance) and *λ*.1se = 0.022 (the most parsimonious model within one SE of the minimum). *λ*.1se was chosen to balance model simplicity and predictive performance. Based on this criterion (*λ*.1se = 0.022), four predictors, including RBC, MCV, MCHC, and RDW‐SD, were retained for the multivariable logistic regression model, Figure 3A,B.

**FIGURE 3:**
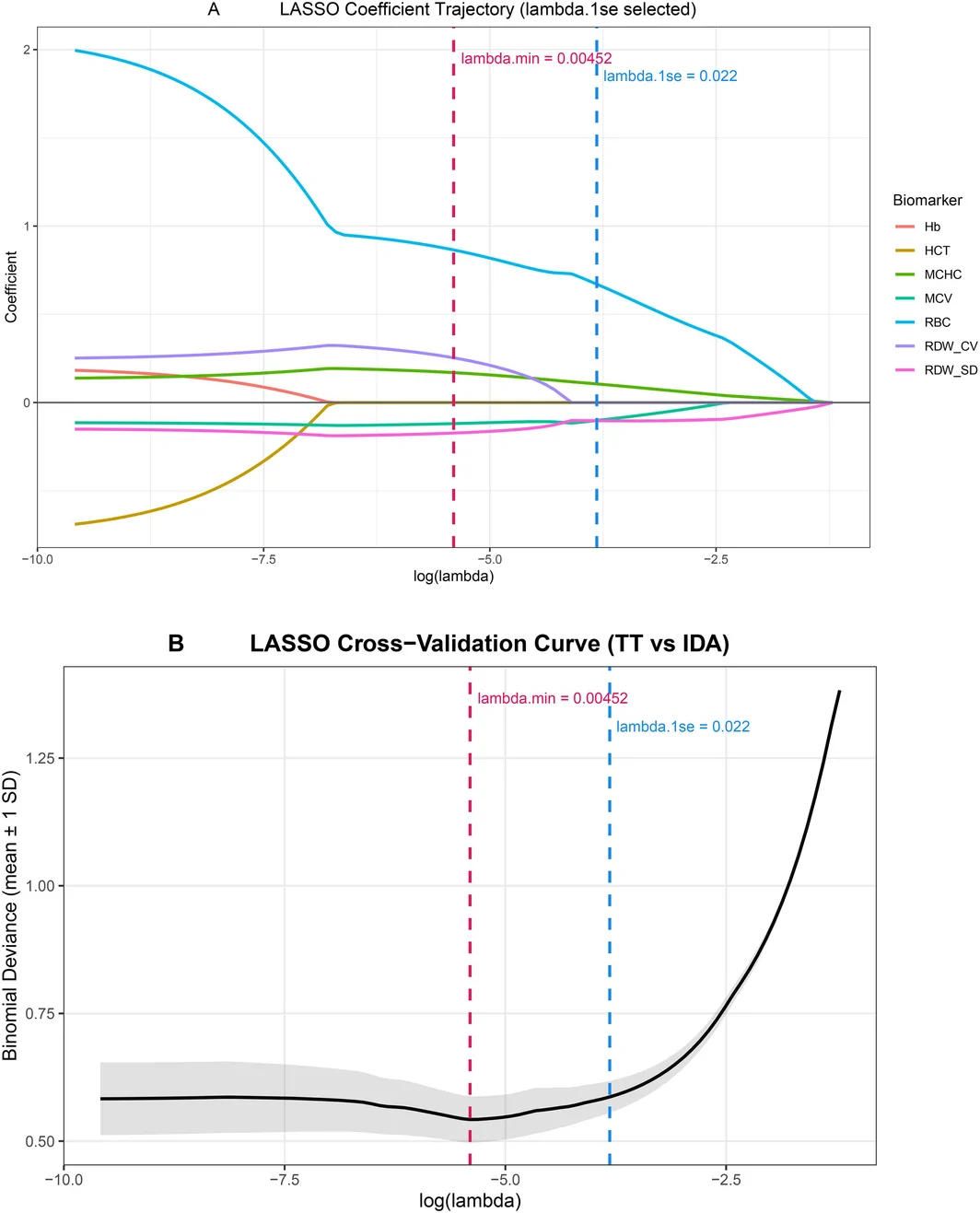
Least absolute shrinkage and selection operator (LASSO) regression analysis for variable selection. (A) LASSO coefficient trajectories of the candidate hematological parameters as a function of log(*λ*). Each curve represented the trajectory of the coefficient for each parameter. The *λ*.min (0.00452) represented the value associated with the lowest deviance and the best predictive performance, whereas *λ*1se (0.022) represented a more simplified model with fewer variables while maintaining comparable accuracy. (B) 10‐fold cross‐validation for tuning parameter selection in the training set. The two vertical dotted lines indicated the optimal *λ* values selected by the minimum criteria (left) and the 1‐standard error (1‐SE) criteria (right). The optimal *λ* (*λ*.1‐SE) was used for final variable selection, retaining RBC, MCV, MCHC, and RDW‐SD.

Four predictors retained by LASSO regression (RBC, MCHC, MCV, and RDW‐SD) were used to construct the multivariable logistic regression model. As shown in Figure 4, RBC (OR = 2.781, 95% CI: 1.831–4.326, *p* < 0.001) and MCHC (OR = 1.195, 95% CI: 1.152–1.246, *p* < 0.001) showed significant positive associations with TT, whereas MCV (OR = 0.825, 95% CI: 0.769–0.878, *p* < 0.001) and RDW‐SD (OR = 0.911, 95% CI: 0.850–0.971, *p* = 0.006) were inversely associated with TT.

**FIGURE 4:**
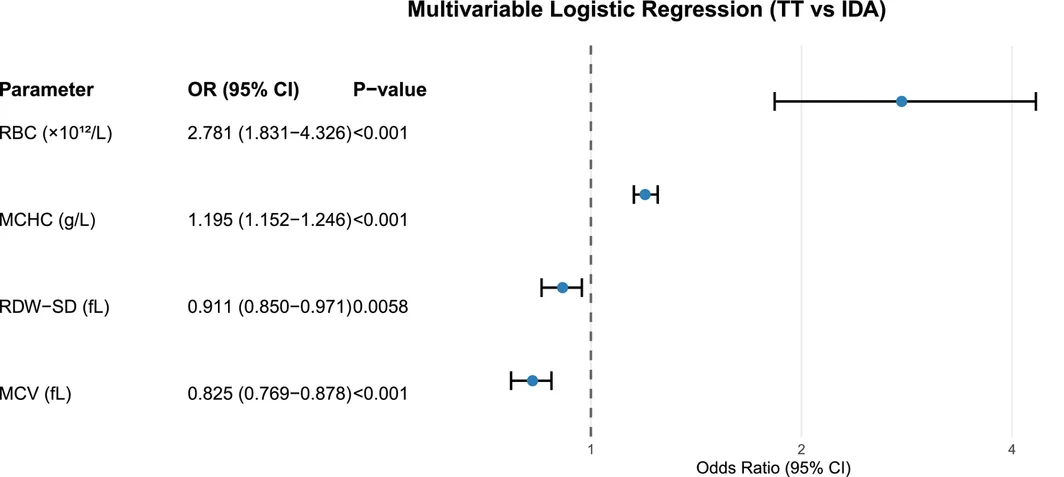
Forest plot displaying odds ratios (ORs) with 95% confidence intervals (CIs) for each parameter in the training cohort. The *x*‐axis was on a log scale, and the dashed vertical line marked OR = 1. RBC and MCHC exhibited positive associations (OR > 1), while MCV and RDW‐SD showed inverse associations (OR < 1) with the incidence of TT. All displayed parameters reached *p* < 0.01.

Base on the four independent predictors included from the multi‐variable model, a nomogram was further constructed to effectively differentiate TT from IDA among MHA patients, Figure 5. To evaluate the calibration of the nomogram, predicted probabilities were plotted against observed outcomes in the test cohort. The diagnostic performance of the model was assessed using the area under the receiver operating characteristic curve (AUC). The nomogram showed favorable discriminating ability, with an AUC of 0.959 (95% CI: 0.944–0.974) in the training set and an AUC of 0.941 (95% CI: 0.909–0.973) in the test set, Figure 6.

**FIGURE 5:**
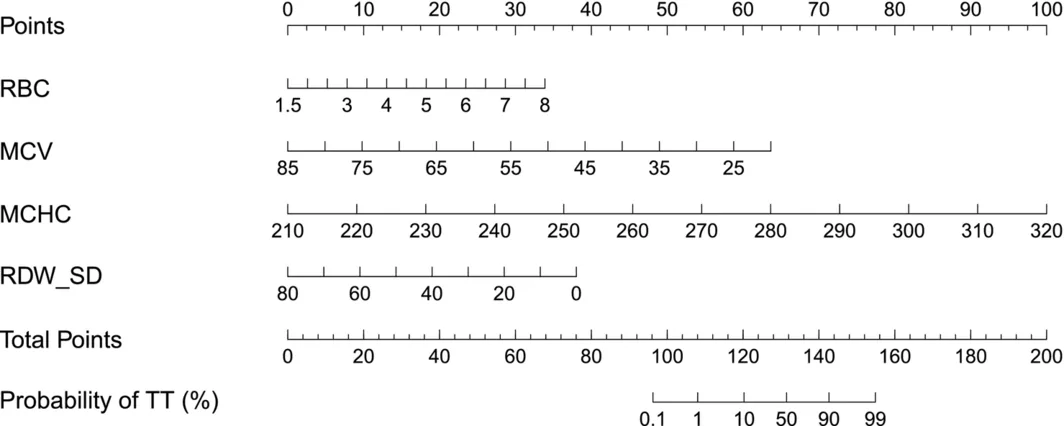
Nomogram for the differential diagnosis of TT and IDA. This predictive nomogram integrated four independent predictors, including red blood cell count (RBC, ×10^12^/L), mean corpuscular volume (MCV, fL), mean corpuscular hemoglobin concentration (MCHC, g/L), and red blood cell distribution width standard deviation (RDW‐SD, fL). To estimate the probability of TT, a vertical line was drawn upward the measured value of each variable to the “Points” axis to obtain the corresponding individual points. All individual scores were summed to obtain the “Total Points,” and a vertical line extended from the total points to the “Probability of TT (%)” axis finally determined the predictive probability of TT. IDA, iron deficiency anemia; TT, thalassemia trait.

**FIGURE 6:**
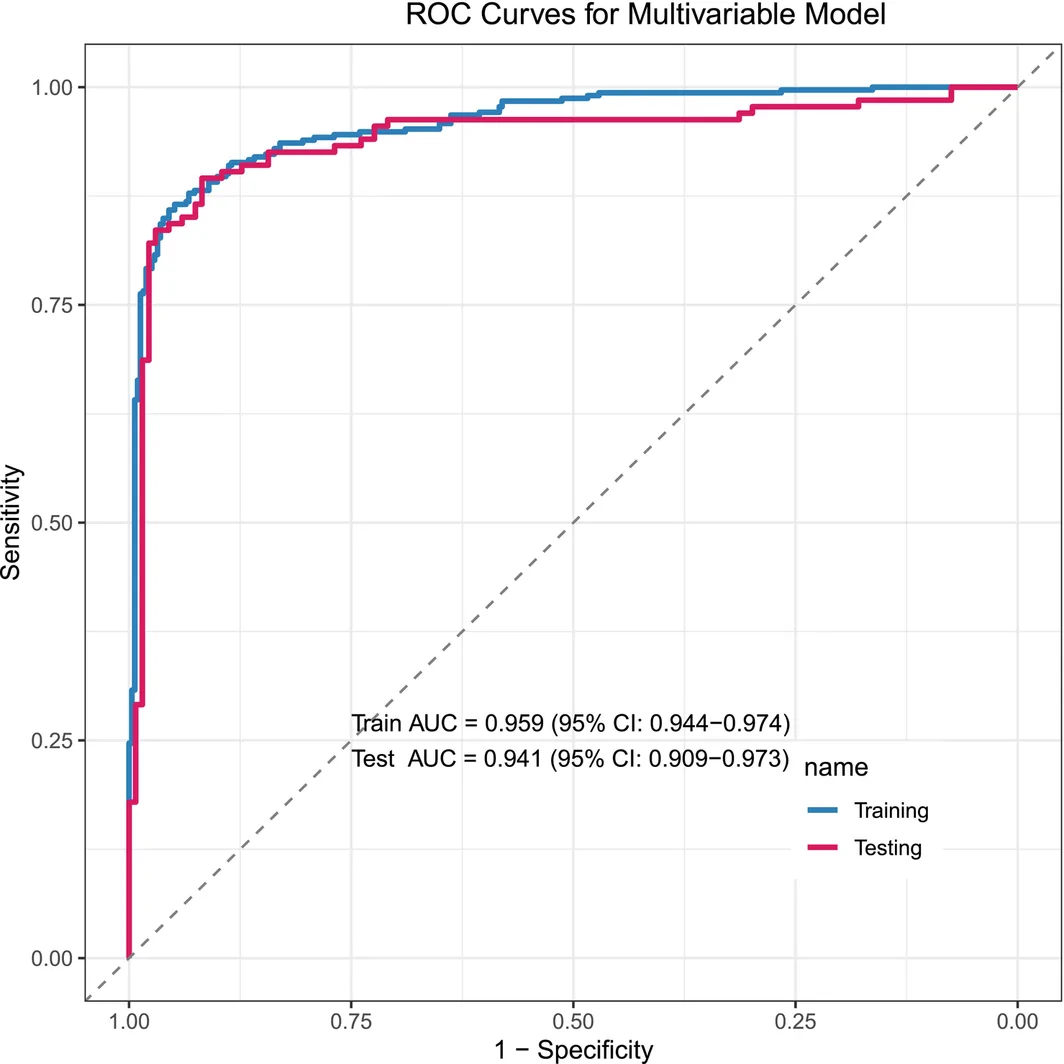
Receiver operating characteristic (ROC) curves for the diagnostic nomogram in the training and testing cohorts. (A) Training cohort (*n* = 624): The nomogram yielded an AUC of 0.959 (95% CI: 0.944–0.974). (B) Testing cohort (*n* = 268): The nomogram achieved an AUC of 0.941 (95% CI: 0.909–0.973). The diagonal dashed line represents the reference line of no discrimination (AUC = 0.5). Both ROC curves demonstrate excellent discriminative ability of the nomogram for distinguishing TT from IDA.

Calibration of the nomogram was assessed in the test cohort, Figure S1. The calibration curves showed satisfactory agreement between predicted and observed probabilities, with a mean absolute error of 0.033, Figure S1. Decision curve analysis (DSA) further demonstrated that the nomogram provided a positive net benefit across a wide range of clinically reasonable threshold probabilities, Figure S2, supporting its potential utility of distinguishing TT from IDA.

In the test cohort, our LASSO‐based nomogram showed superior discriminatory performance compared with other indices for differentiating TT from IDA, including Mentzer (cut‐off = 13) [19], Shine and Lal (cut‐off = 1530) [20] and Green and King (cut‐off = 65) indices [21]. It achieved the highest AUC (0.94), accuracy (90.1%), and Youden index (0.81) with favorable sensitivity (90.0%) and specificity (91.8%), Table 4.

**TABLE 4:** Diagnostic performance of the LASSO‐based nomogram compared with other indices.

Index	LASSO nomogram	Mentzer index	Shine & Lal index	Green & King index
Cutoff	0.55	13	1530	65
Sensitivity	0.9	0.69	0.93	0.99
Specificity	0.92	0.89	0.13	0
PPV	0.92	0.86	0.52	0.5
NPV	0.9	0.74	0.65	0
Accuracy	0.901	0.79	0.53	0.5
AUC	0.94	0.84	0.61	0.88
Youden	0.81	0.57	0.06	0
TP	120	92	125	133
FP	11	15	117	134
FN	14	42	9	1
TN	123	119	17	0

*Note:* Data were presented as performance efficacy at the optimal cut‐off value determined by the Youden index for the nomogram, and at the conventional cut‐off values for the classical indices. The testing cohort comprised 134 thalassemia trait (TT) patients and 134 iron deficiency anemia (IDA) patients.

Abbreviations: AUC, area under the curve; FN, false negative; FP, false positive; NPV, negative predictive value; PPV, positive predictive value; TN, true negative; TP, true positive; Youden, Youden's index.

## 4 Discussion

In this study, we systematically characterized the genetic landscape of thalassemia trait (TT) among individuals from Northern China and constructed a LASSO‐based nomogram to effectively discriminate TT from iron deficiency anemia (IDA) using four routine hematological parameters. Our findings revealed a distinct β‐TT‐dominant genotype spectrum in Northern China, underscoring the limitations of fixed‐cutoff discriminative indices for cross‐population applications, and presented a convenient, population‐specific, data‐driven predictive tool with satisfactory diagnostic performance in routine clinical settings.

The predominance of β‐TT (54.15%) over α‐TT (43.36%) in our population significantly differed from the well‐documented pattern in Southern China, where α‐TT constitutes the majority of TT cases [5, 22, 23, 24]. This finding was consistent with a previous large‐scale genotype analysis of Northern Chinese populations [10]. Such epidemiological features may be attributable to both historical endemicity and population migration. One prior study reported that 82.7% of TT carriers in Northern China were migrants originating from Southern provinces [25], suggesting that the current mutation spectrum in Northern China was far from a purely indigenous profile. Notably, the three most prevalent β‐TT mutations detected among our Northern subjects (*HBB:c.316‐197C>T het, HBB:c.124_127delTTCT het, HBB:c.52A>T het*) were also common in Southern China, indicating a shared genetic basis [26]. Nevertheless, their relative frequencies differ with *HBB:c.124_127delTTCT het* ranking higher in some Southern reports [7, 27, 28]. Collectively, these observations further supported the existence of regional heterogeneity in the allele‐frequency distribution of TT.

Beyond common mutations, two cases of *HKαα* were identified in our cohort, further highlighting the genetic complexity of TT in Northern China. The *HKαα* variant, an α‐globin gene rearrangement originally reported in Southern populations, has been reported at frequencies ranging from 0.33% to 2.0% in Southern China [29]. Although detected at a low frequency (1.0% of α‐TT cases in our study), *HKαα* underscored the genetic diversity of TT in Northern China beyond well‐known α‐ and β‐globin mutations [30]. Nevertheless, *HKαα* may be prone to being overlooked by routine genotyping methods due to its mild clinical anemic manifestations [30, 31]. Its identification in our Northern cohort emphasized the necessity of comprehensive molecular screening, even in regions traditionally regarded as low‐prevalence areas for TT.

The above‐described genetic complexity in this study underpinned our model‐building strategy for differential diagnosis. Existing models reported for TT‐IDA differentiation were either α‐TT‐focused nomograms developed based on Southern China populations or complex machine learning algorithms [3, 32]. These tools lacked applicability to the distinct TT profile of Northern China and were poorly suited for primary‐care settings. Herein, we constructed a LASSO‐based nomogram incorporating RBC, MCV, MCHC, and RDW‐SD based on Northern China residents, which achieved excellent discriminative efficiency in the independent testing patient group (AUC = 0.94), with favorable sensitivity (90.0%) and specificity (92%). The multivariate forest plot further demonstrated that RBC and MCHC exerted positive predictors for TT, with corresponding odds ratios of 2.781 and 1.195, respectively, while MCV and RDW‐SD served as negative independent predictors. These differences may have resulted from distinct erythropoietic disturbances underlying the two disorders. Specifically, congenital globin‐chain defects trigger compensatory erythroid proliferation in TT patients, leading to markedly higher RBC counts compared with those with IDA. On the contrary, insufficient iron supply impairs hemoglobin synthesis in IDA, which concurrently reduces erythrocyte volume and produces substantial erythrocyte‐size heterogeneity (higher RDW‐SD). These intergroup discrepancies were well reflected in the baseline hematologic profiles of the training and internal‐validation cohorts.

Several recently developed approaches have been reported to differentiate TT from IDA with variable diagnostic performance, including the TT@MHA algorithm (AUC = 0.942), the Multi‐Thal classifier (AUC = 0.941), the Basrah Score (accuracy = 96.7%) and a Light GBM‐based classifier (AUC = 0.992) [32, 33, 34, 35]. Additionally, an RBC‐parameter‐based logistic‐nomogram model was also developed in Southern Fujian (AUC = 0.95), in which RBC, MCH and MCHC were identified as independent predictive indicators for TT‐IDA differentiation [3]. Notably, unlike the Southern model, which excluded MCV and RDW‐SD, our findings identified MCV and RDW‐SD as informative predictors, reflecting population‐specific hematological features. Nevertheless, most existing diagnostic tools were primarily derived from the Southern Chinese cohort and have not been validated in Northern Chinese populations, who carry distinct predominant TT genotypes. Such inconsistent performance across different diagnostic approaches, together with the regional genetic heterogeneity observed in our subjects, strongly highlighted the necessity of population‐specific validation.

Despite the availability of these novel diagnostic approaches, commonly used classical hematological indices, such as Mentzer, Shine and Lal, and Green and King indices [19, 20, 21], remain widely employed in primary‐care hospitals owing to their simplicity. Nevertheless, no single index consistently outperformed others across different populations [36, 37, 38]. Such variability among both conventional indices and machine‐learning‐based diagnostic tools underscored a fundamental principle: that is, population‐specific calibration is critical. Thus, our nomogram, built on populations from Northern China, exhibited superior discriminatory performance compared with routine indices and could effectively fill this clinical‐practice gap.

When directly comparing our nomogram with the Mentzer, Shine and Lal, and Green and King indices using their conventional cutoffs, our nomogram outperformed the other three indices via the Youden index with superior sensitivity and specificity. Notably, the Shine and Lal index had high sensitivity (93.3%) but extremely low specificity (12.7%), while the Green and King index exhibited extremely low specificity at its conventional cutoff—reinforcing the concern that fixed‐threshold rules derived from other populations may not be suitable for Northern China. A previous study demonstrated that the Mentzer index (MCV/RBC) relies on divergent red‐blood‐cell count patterns across different hematologic conditions [19]. β‐TT drives compensatory erythroid proliferation, resulting in elevated RBC counts accompanied by reduced MCV. By contrast, IDA impairs erythropoiesis, leading to a decrease in RBC and MCV. For α‐TT, however, the compensatory proliferative response is less pronounced, accounting for the lower diagnostic accuracy of the Mentzer index for α‐TT. This biological constraint underscores the necessity of multi‐parameter diagnostic strategies.

However, several limitations of the present study should be acknowledged. First, the sample size of this study is relatively small and all subjects enrolled from one single center without external validation, which may introduce bias when evaluating the predictive efficacy of the model constructed. Second, our cohort comprised predominantly Han Chinese adults from Northern China. Accordingly, the generalizability of the nomogram to other ethnic groups, pediatric populations and pregnant women remains unclear. Third, despite its favorable diagnostic predictive efficacy, LASSO regression suffers inherent limitations in variable selection stability and its outputs should not be overinterpreted. Furthermore, the nomogram may exhibit diminished discriminatory performance among patients with borderline hematological profiles, such as individuals with MCV between 70 and 75 fL or RDW‐CV between 15% and 17%, where hematological features of TT and IDA overlap most substantially. For such equivocal cases, we recommend confirmatory testing, including genetic analysis and iron studies, to establish a definitive diagnosis. Additionally, patients with concurrent hematological disorders were excluded from this study. Accordingly, the applicability of this nomogram to patients with combined anemia or other hematological conditions remains unclear and warrants further investigation. Finally, the clinical utility of the nomogram has not been prospectively evaluated in real‐world settings. Multi‐center, prospective studies are therefore warranted to validate and refine the nomogram prior to its future clinical application.

In summary, this study provided novel insights into the genetic characteristics of TT in Northern China and presented a simple, effective and region‐appropriate diagnostic nomogram to differentiate TT from IDA. By integrating population‐specific genetic data with accessible clinical hematological parameters, our findings offer a practical tool to improve the diagnosis and treatment of anemia in Northern China and underscored the significance of customizing diagnostic models to regional and demographic contexts.

## Author Contributions

Yan Long took charge of all the work and participated in its design. Wen‐jie Yu and Feng‐qi Zhang collected most part of data, drafted the manuscript and contributed to its design. Wen‐jie Yu and Jia‐feng Niu undertook the data analysis. Rui Ma, Guang‐ping Li, and Yin‐ting Ma carried out most of the experiments. All the authors approved the final draft submitted.

## Funding

This work was supported by National Natural Science Foundation of China (82002214).

## Ethics Statement

This study was in accordance with the Declaration of Helsinki and was approved by the ethical committee of Peking University People's Hospital. All original data involved in this study were managed by Peking University People's Hospital. As a retrospective study, we only collected clinical information of patients, without interfering with clinical treatment or bringing any physiological risks to enrolled subjects. Our team has taken strict measures to protect patient privacy and prevent personal information disclosure. Informed consent is obtained for retrospective studies before sample collection.

## Conflicts of Interest

The authors declare no conflicts of interest.

## Supporting information


**Table S1:** Baseline characteristics of train set.

**Table S2:** Baseline characteristics of test set.

**Figure S1:** Calibration curve of the nomogram for predicting thalassemia trait in the testing set (*n* = 268). Calibration curve assessing the agreement between predicted probabilities and observed outcomes in the independent testing cohort (*n* = 268). The *x*‐axis represents the predicted probability of TT generated by the nomogram, and the *y*‐axis represents the actual observed proportion of TT. The dashed diagonal line indicates perfect calibration (ideal). The apparent and bias‐corrected curves were shown, with the latter generated by 1000 bootstrap resamples to reduce overfitting optimism. The shaded area represents the 95% CIs. The MAE of 0.033 indicates good agreement between predicted and observed probabilities. CIs, confidence intervals; MAE, mean absolute error; TT, thalassemia trait.

**Figure S2:** Decision curve analysis evaluating the clinical utility of the nomogram in the testing cohort (*n* = 268). The *x*‐axis represents the threshold probability, and the *y*‐axis represents the net benefit. The blue curve shows the net benefit of the nomogram across a range of clinically reasonable threshold probabilities. The gray lines represent the “treat‐all” and “treat‐none” strategies. The nomogram demonstrated a positive net benefit across a wide range of threshold probabilities, supporting its clinical utility. DCA, decision curve analysis.

## Data Availability

The data sets used or analyzed during the current study are available from the corresponding author on reasonable request.
