# Supplementary material for: Genetic and Clinical Characteristics of Thalassemia Trait and Construction of LASSO‐Based Model for Its Differentiating From Iron Deficiency Anemia in Northern China
Source: J Clin Lab Anal. 2026 Oct 26:e70386. Online ahead of print. doi: 10.1002/jcla.70386 (PMC13710943; doi:10.1002/jcla.70386)
Supplement: Supplementary file 1 [file JCLA-9999-e70386-s002.pdf]

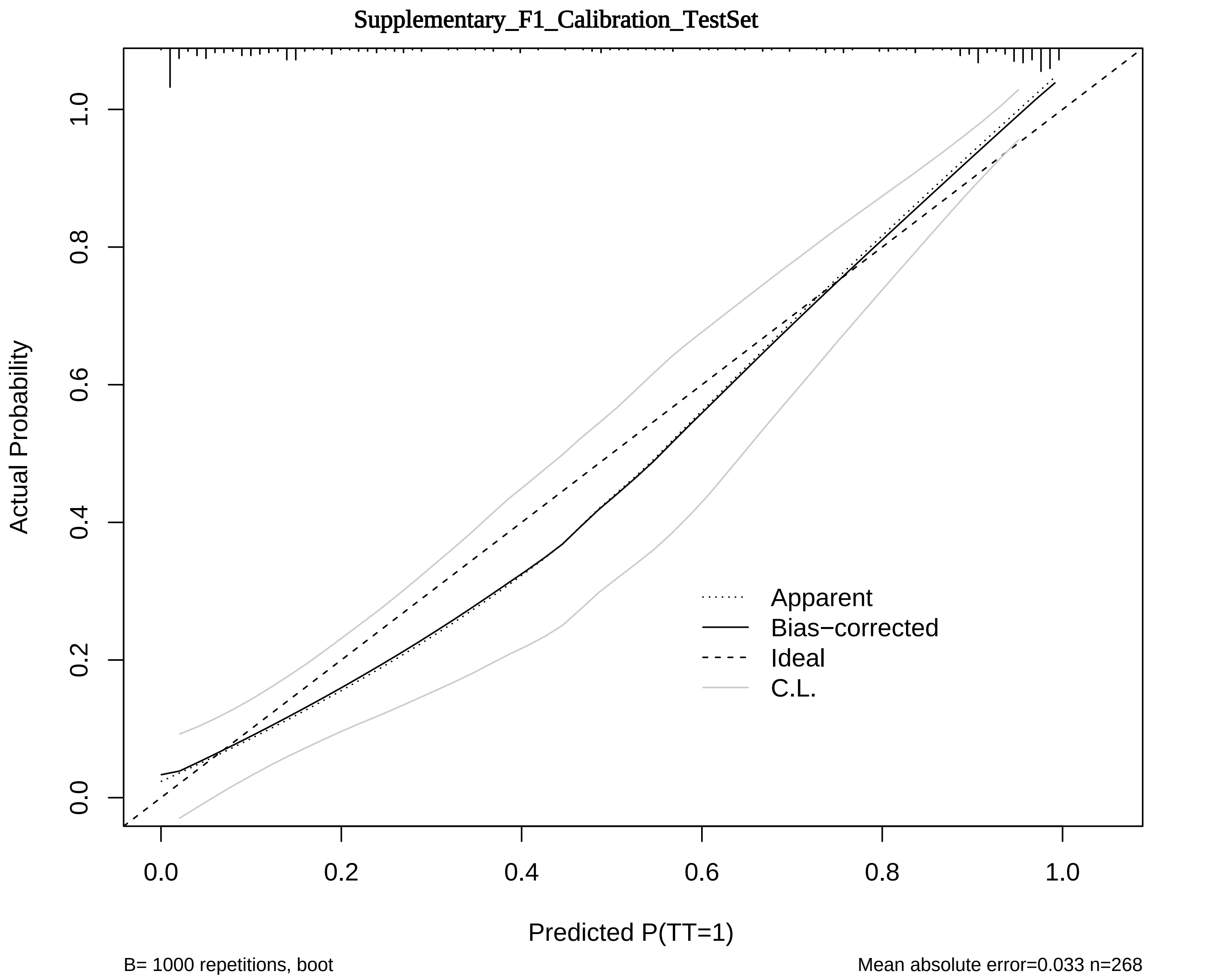
Supplementary_F1_Calibration_TestSet
Actual Probability
0.0
0.2
0.4
0.6
0.8
1.0
Predicted P(TT=1)
Apparent
Bias−corrected
Ideal
C.L.
B= 1000 repetitions, boot
Mean absolute error=0.033 n=268
