# Supplementary material for: Genetic and Clinical Characteristics of Thalassemia Trait and Construction of LASSO‐Based Model for Its Differentiating From Iron Deficiency Anemia in Northern China
Source: J Clin Lab Anal. 2026 Oct 26:e70386. Online ahead of print. doi: 10.1002/jcla.70386 (PMC13710943; doi:10.1002/jcla.70386)
Supplement: Supplementary file 2 [file JCLA-9999-e70386-s003.pdf]

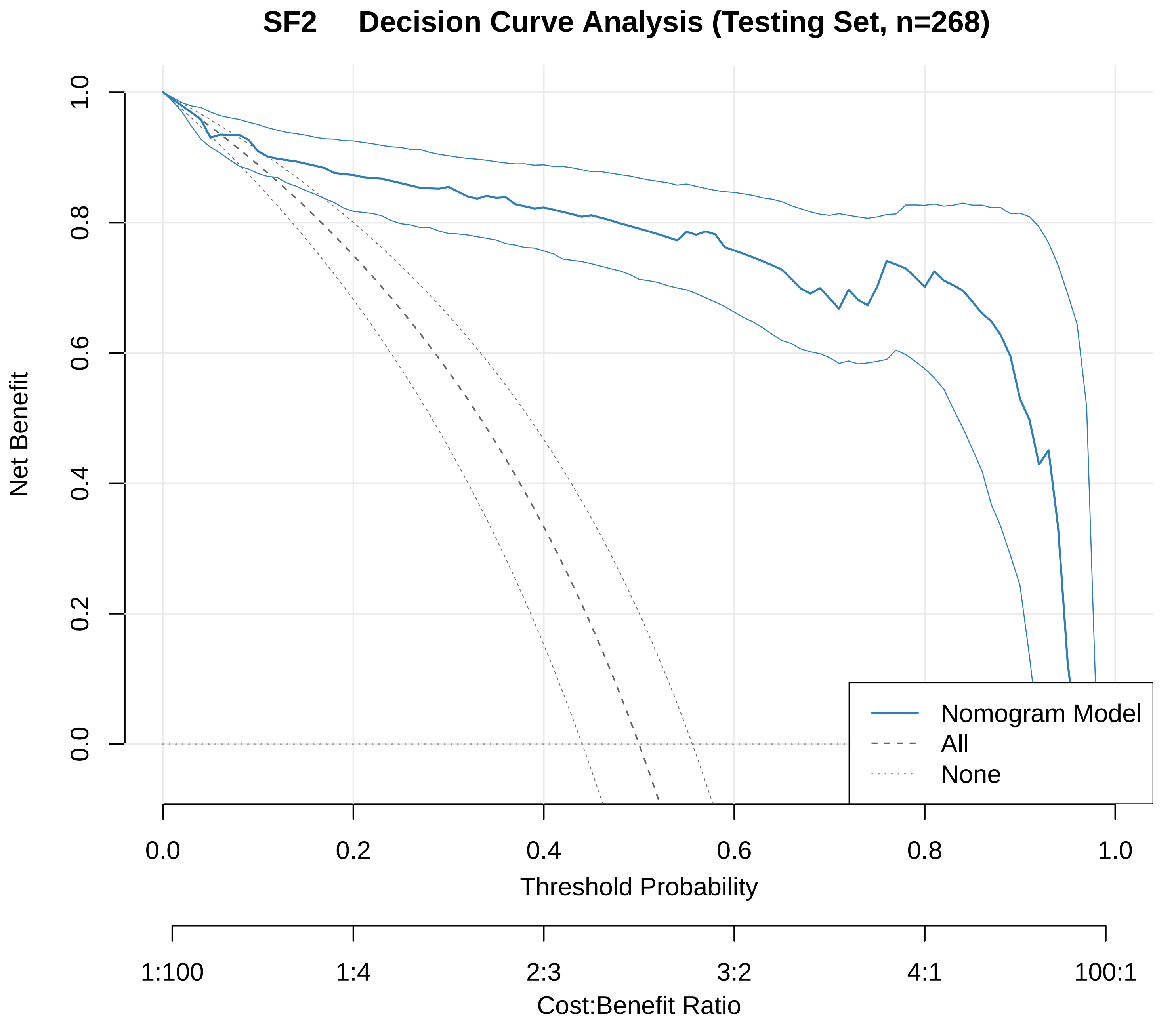
SF2 Decision Curve Analysis (Testing Set, n=268)
Net Benefit
1.0
0.8
0.6
0.4
0.2
0.0
Nomogram Model
All
None
0.0
0.2
0.4
0.6
0.8
1.0
Threshold Probability
1:100
1:4
2:3
3:2
4:1
100:1
Cost:Benefit Ratio
