# Supplementary material for: Genetic and Clinical Characteristics of Thalassemia Trait and Construction of LASSO‐Based Model for Its Differentiating From Iron Deficiency Anemia in Northern China
Source: J Clin Lab Anal. 2026 Oct 26:e70386. Online ahead of print. doi: 10.1002/jcla.70386 (PMC13710943; doi:10.1002/jcla.70386)
Supplement: Supplementary file 3 [file JCLA-9999-e70386-s001.docx]

**Table S1 Baseline characteristics of train set**

| **Variable** | **Total(n=624)** | **TT(n=312)** | **IDA(n=312)** | ***P*-value** |
| --- | --- | --- | --- | --- |
| Age，Median(Q1,Q3) | 32.0(28.0,41.00) | 32.0(28.0，41.0) | 32.0(28.0,41.0) | 0.021 |
| RBC(×10^12^/L),Median(Q1,Q3) | 4.8(4.26,5.55) | 5.5（4.9,6.1） | 4.4(4.0,4.9) | <0.001 |
| HB(g/L),Median(Q1,Q3) | 103.0(88.0,116.0) | 112.0（102.0，124.0） | 92.0(75.3,104.0) | <0.001 |
| HCT(%),Median(Q1,Q3) | 33.9(30.1,37.8) | 36.6（32.8，40.3） | 31.7(27.7,35.0) | <0.001 |
| MCV(fL),Median(Q1,Q3) | 68.9(64.9,73.8) | 66.9（63.8，70.0） | 71.7(67.4,75.8) | <0.001 |
| MCH(pg),Median(Q1,Q3) | 20.6(19.3,22.0) | 20.1(19.6，21.5) | 20.8(18.5,22.5) | 0.218 |
| MCHC(g/L),Median(Q1,Q3) | 301.0(288.0,310.0) | 308.0(301.3，312.0) | 289.0(273.0,299.0) | <0.001 |
| RDW-CV(%),Median(Q1,Q3) | 16.9(15.5,18.6) | 16.1(15.1,17.2) | 17.7(16.2,19.9) | <0.001 |
| RDW-SD(fL),Median(Q1,Q3) | 40.5(36.1,45.3) | 36.2(34.5，38.4) | 44.8(42.5,47.8) | <0.001 |

IDA, iron deficient anemia; TT, thalassemia trait; RBC, red blood cell; HB, hemoglobin; HCT, hematocrit; MCV, mean corpuscular volume; MCH, mean corpuscular hemoglobin; MCHC, mean corpuscular hemoglobin concentration; RDW-CV, red blood cell distribution width coefficient of variation; RDW-SD, red blood cell distribution width standard deviation.
