# Supplementary material for: Genetic and Clinical Characteristics of Thalassemia Trait and Construction of LASSO‐Based Model for Its Differentiating From Iron Deficiency Anemia in Northern China
Source: J Clin Lab Anal. 2026 Oct 26:e70386. Online ahead of print. doi: 10.1002/jcla.70386 (PMC13710943; doi:10.1002/jcla.70386)
Supplement: Supplementary file 4 [file JCLA-9999-e70386-s004.docx]

| **Table S2 Baseline characteristics of test set** | | | | | |
| --- | --- | --- | --- | --- | --- |
| **Variable** | **Total(n=268)** | **TT(n=134)** | **IDA(n=134)** | ***p*-value** |  |
| Age,Median(Q1,Q3) | 33.0(28.0,43.0) | 33.0(27.8,43.3) | 33.0(28.0,43.3) | 0.991 |  |
| RBC(×10^12^/L),Median(Q1,Q3) | 4.8(4.2,5.8) | 5.6(4.9,6.3) | 4.4(3.9,4.8) | <0.001 |  |
| HB(g/L),Median(Q1,Q3) | 102.0(85.0,116.0) | 114.0(102.8,128.0) | 92.5(74.4,101.3) | <0.001 |  |
| HCT(%),Median(Q1,Q3) | 34.3(29.7,38.2) | 37.1(32.8,42.4) | 31.9(27.5,34.9) | <0.001 |  |
| MCV(fL),Median(Q1,Q3) | 68.0(64.9,74.1) | 66.8(63.7,69.8) | 71.9(66.3,77.5) | <0.001 |  |
| MCH(pg),Median(Q1,Q3) | 20.7(19.1,22.1) | 20.6(19.5,21.5) | 20.8(18.0,23.1) | 0.949 |  |
| MCHC(g/L),Median(Q1,Q3) | 300.5(286.0,310.0) | 308.0(301.8,312.3) | 289.0(273.5,300.0) | <0.001 |  |
| RDW-CV(%),Median(Q1,Q3) | 17.0(15.4,18.7) | 16.4(15.1,17.9) | 17.7(16.2,19.9) | <0.001 |  |
| RDW-SD(fL),Median(Q1,Q3) | 41.0(35.8,45.2) | 35.8(34.4,38.5) | 44.6(42.2,47.7) | <0.001 |  |

IDA, iron deficient anemia; TT, thalassemia trait; RBC, red blood cell; HB, hemoglobin; HCT, hematocrit; MCV, mean corpuscular volume; MCH, mean corpuscular hemoglobin; MCHC, mean corpuscular hemoglobin concentration; RDW-CV, red blood cell distribution width coefficient of variation; RDW-SD, red blood cell distribution width standard deviation.
